# Selection of Pneumatic Reduction in Invagination Treatment and the Factors Affecting the Success of This Method

**DOI:** 10.7759/cureus.5928

**Published:** 2019-10-16

**Authors:** Mustafa Erman Dörterler, Osman Hakan Kocaman

**Affiliations:** 1 Pediatric Surgery, Harran University Faculty of Medicine, Şanlıurfa, TUR

**Keywords:** intussusception, pneumatic reduction, ultrasonography, fluoroscopy

## Abstract

Objective

To retrospectively evaluate the success rates of fluoroscopy-guided pneumatic reduction in children with intussusception and to determine the risk factors and patient characteristics affecting the success of this method.

Materials and methods

Retrospective evaluation was made of a total of 183 children diagnosed with ultrasonography (USG) and treated in the pediatric surgery clinic between August 2010 and December 2017. Data related to gender, age (month), date of presentation, invaginated segment localizations and treatment modalities were retrieved from the patient files.

In children who underwent pneumatic reduction, surgical success was determined as the visualization of air flow through the small intestine on fluoroscopy and patients who received reduction were followed up in our clinic. No progress of the air given or failure to see the air flow to the small intestine despite some progression of the air was evaluated as failure and surgical procedure was started in 39 patients. All patients were followed up at the hospital for at least 24 to 48 hours after the procedures.

Patients with perforation, peritonitis, vital instability, or general condition disorder during presentation at our clinic (n = 29) were directly admitted for surgery.

Results

The study included a total of 183 children, comprising 116 (63.4%) males and 67 (36.6%) females with a mean age of 61.3 ± 34.3 months. While mesenteric lymphadenitis (n = 7) was determined as the most common lesion in cases where a pathologic lead point was detected (7.6%), Meckel’s diverticulum was observed in four patients, polyps in two patients, and an involvement associated with Henoch Schönlein Vasculitis in one patient. Pneumatic reduction procedure was applied in 154 (84.1%) patients and successful results were obtained in 115 (74.7%) patients, while surgical intervention was required in 39 (25.3%) patients.

While frequency of admission to direct surgery following the failure of pneumatic reduction in children under the age of two years was determined to be higher than the frequency of healing, the successful pneumatic reduction and admission to direct surgery rates in children between the ages of 0-4 years was found to be significantly higher than those in other age groups (p < 0.001). The pneumatic reduction success rate was determined to decrease significantly in children aged ≥6 years (p < 0.001). The mean hospitalization duration of the patients who underwent surgery after pneumatic reduction (five days) was determined to be significantly longer compared to that of the patients who underwent direct surgery and for whom a successful pneumatic reduction was ensured (p = 0.001 and p = 0.008, respectively).

Conclusion

Fluoroscopy-guided pneumatic reduction has a high success rate and is still one of the first option methods in the treatment of idiopathic intussusception. The application of the method under operating room conditions is more appropriate for patient safety. It is considered that the non-operative reduction success may increase with the detailed evaluation of intussusception cases determined to have pathologic lead points in children aged <2 years and >6 years.

## Introduction

Intussusception is a disease that is very common in infancy and early childhood (predominant in the male gender) and causes acute abdomen (approximate prevalence 1-4/2000) [1-3]. The clinical manifestations of the disease include persistent crying episodes, palpable abdominal mass, abdominal pain, abdominal distention, and viscous and bloody stool. Intussusception can occur for many different etiological reasons and is known to be most commonly idiopathic [2,4]. Pathologic lead point-induced intussusception cases account for 6-10% of all cases and are frequently caused by Meckel’s diverticulum, intestinal polyps, inflammatory bowel diseases, and benign or malignant intestinal tumors [5,6].

Cases of intussusception that are not treated at the right time and with the right method can result in death. Although intussusception can be treated with minimally invasive methods when diagnosed early, complications such as intestinal necrosis, perforation and sepsis may develop in patients who are diagnosed late.

Operative and non-operative treatment methods may be preferred for patients diagnosed with intussusception. In the absence of necrosis and perforation in idiopathic intussusception, it is recommended that the first non-operative option be pneumatic or hydrostatic reductions. A non-operative reduction may be performed by hydrostatic or pneumatic pressure enema under ultrasound or fluoroscopy. Although they differ significantly between health centers, the success rates of these treatment methods vary between 45-94%. The fluoroscopy-guided pneumatic reduction procedure is currently used as the first-line non-operative reduction method in many clinics. This method is a very useful, repeatable, inexpensive and accessible technique that can be easily used for early diagnosis of intussusception and evaluation of the success of the treatment. Patients should receive ultrasonography (USG)-guided pneumatic reduction and should be taken for emergency laparotomy if the treatment fails. The main disadvantage of sonography is the need for an experienced radiologist. Emergency laparotomy is the first option treatment in case of suspicion of necrosis, with imaging findings suggesting the presence of a pathologic lead point, perforation, and hemodynamic instability.

The aim of this study was to retrospectively evaluate the success rates of fluoroscopy-guided pneumatic reduction in children with intussusception and to provide information about the risk factors and patient characteristics affecting the success of this method.

## Materials and methods

Patients

In this study, retrospective evaluation was made of a total of 183 children diagnosed through USG and treated in the pediatric surgery clinic between August 2010 and December 2017. The gender, age (months), date of presentation, invaginated segment localizations and treatment modalities of the cases were retrieved from the patient files.

Pneumatic reduction and surgery

High-resolution ultrasound (GE® LOGIQ P9) was used by at least one experienced radiologist to visualize intussusception in children who were admitted to the operating room with the diagnosis of intussusception. A total of 29 patients with perforation, peritonitis, vital instability, and general condition disorder on presentation at the clinic were directly admitted for surgery.

A total of 154 patients diagnosed with intussusception and without contraindications for pneumatic reduction underwent sedation (to be prepared for conditions such as perforation and unsuccessful reduction during the process) and were applied with fluoroscopy-guided (EOC Fluostar® 7900) pneumatic reduction under operating conditions. In our clinic, fluoroscopy-guided pneumatic reduction (FGPR) is routinely used in the treatment of intussusception, except in cases of perforation, peritonitis, vital instability, and general condition disorder.

**Figure 1:**
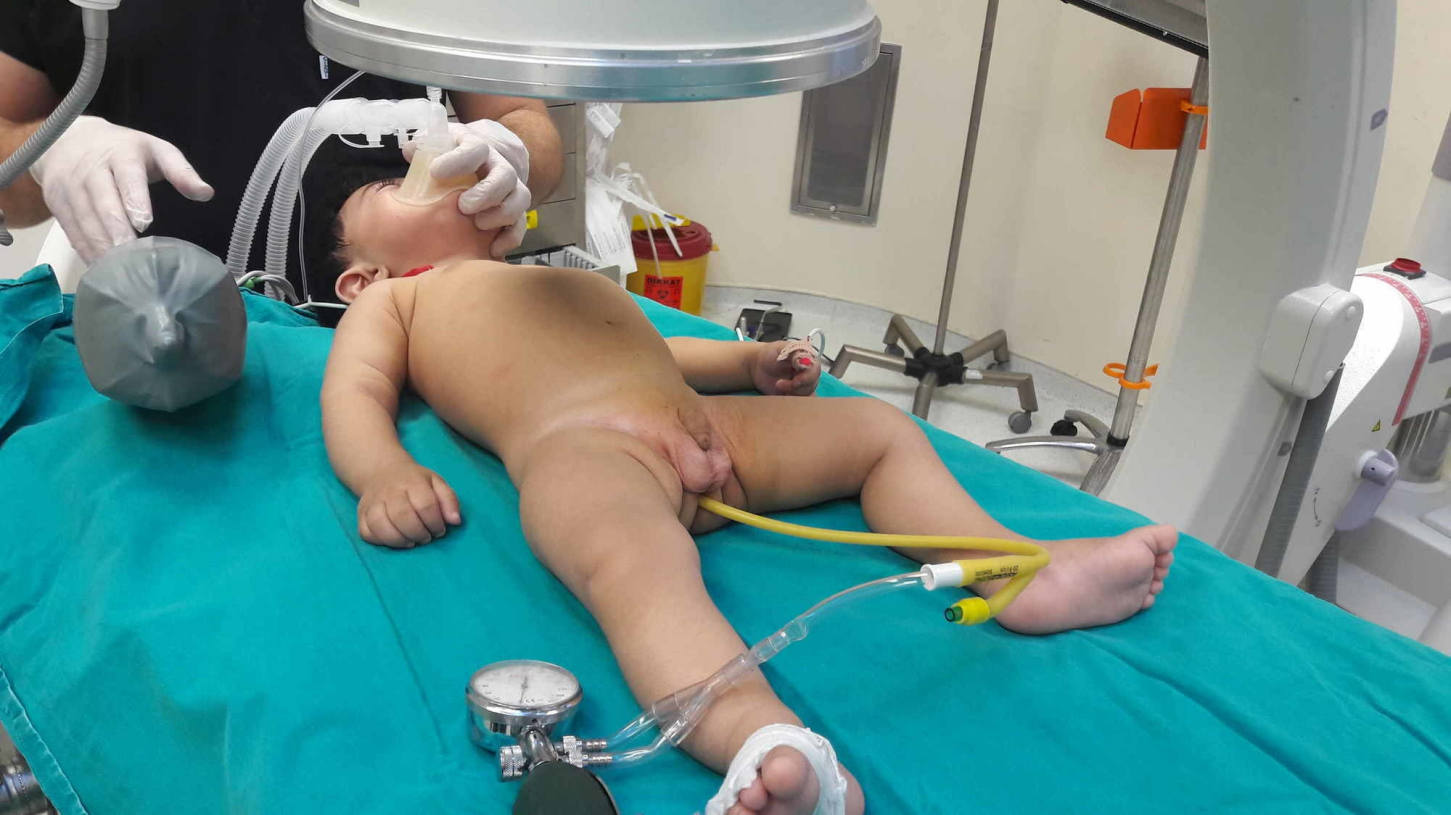
Fluoroscopy-guided pneumatic reduction under operating room conditions in a child diagnosed with intussusception

In the pneumatic reduction procedure, a 14-22 Fr Foley catheter was placed in the rectum with the child in the supine position, and the air was administered in such a way as not to exceed 120 mmHg (Figure 1).

**Figure 2:**
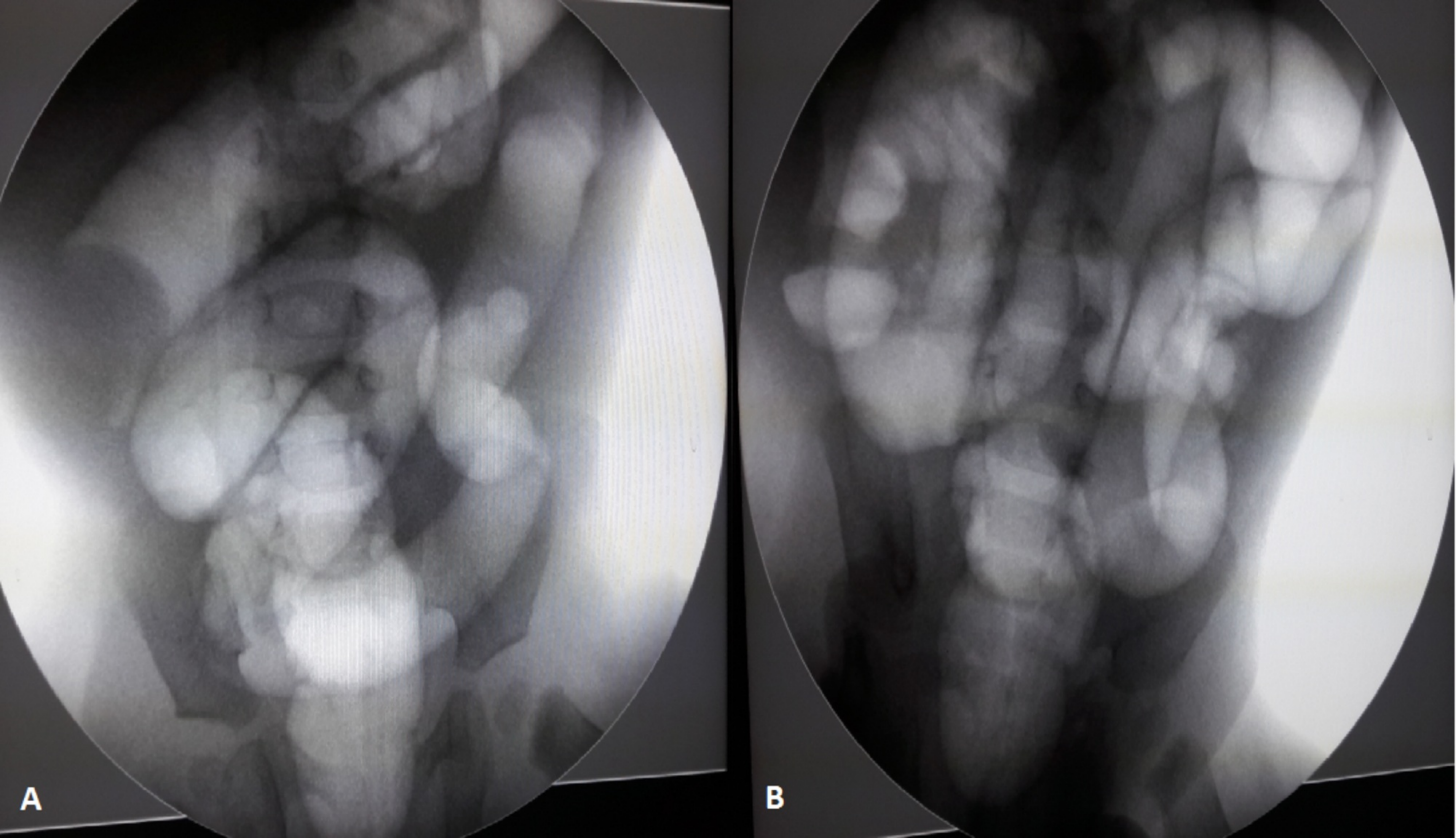
(A) Ileocolic intussusception segment shown under fluoroscopy. (B) Appearance after a successful pneumatic reduction under fluoroscopy.

Under fluoroscopy, it was decided that the procedure was successful when the passage of air into the small intestine was seen, and the case was then followed up in our clinic (Figure 2).

No progress of the air given or failure to see the air flow to the small intestine despite some progression of the air was evaluated as failure and the surgical procedure was started in 39 patients.

All patients were given midazolam at a dosage of 0.1 mg/kg for premedication. Experienced surgeons from the pediatric surgery department were also present in the operating room. All patients were followed up for at least 24-48 hours in the hospital after the procedures.

Statistical analysis

SPSS 25.0 software (IBM Corporation, Armonk, NY, USA) was used for the analysis of the variables. Conformity of the data to normal distribution was evaluated with the Shapiro-Wilk test and variance homogeneity with the Levene test.

One-way ANOVA (Robust Statistic: Brown-Forsythe) was used for the comparison of the groups of surgery after unsuccessful pneumatic reduction, direct surgery and successful pneumatic reduction according to the WBC quantitative variable, and the Kruskal-Wallis H tests were used to compare age, duration of surgery, length of hospital stay, CRP, AST, ALT, urea, creatinine, HCT, and HGB variables. The Dunn’s test was applied for the comparison of the results which were found to be significant. The One-Sample Chi-Square and Binomial tests were used to determine the homogeneity of the variables of the procedure performed, month and season of presentation, intussusception localization, pathologic lead point, and gender.

The Pearson Chi-Square and Fisher-Freeman-Holton tests were used with the Monte Carlo Simulation technique for the comparison of the groups of surgery after unsuccessful pneumatic reduction, direct surgery and successful pneumatic reduction according to the variables of season, intussusception localization, pathologic lead point, gender and categorized age, and the column ratios were compared with each other and expressed according to Benjamini-Hochberg corrected p-value results. Multinominal logistic regression analysis was applied to determine the cause-effect relationship of unsuccessful pneumatic reduction, direct surgery and successful pneumatic reduction response variables with the variables of gender, age, duration of surgery and length of hospital stay. Quantitative variables were shown as mean ± standard deviation (SD) and median (Minimum / Maximum) values, and categorical variables as number (n) and percentage (%) in the tables. The variables were analyzed at 95% confidence level and a value of p < 0.05 was accepted as statistically significant.

## Results

Evaluation was made of a total of 183 children, comprising 116 (63.4%) males and 67 (36.6%) females with a mean age of 61.3 ± 34.3 months (p < 0.001) (Table 1).

**Table 1:** General characteristics of the cases included in the study ^1^ One-sample Chi-square test ^2^ One-sample binomial test ^A, B, C^ Significant according to the relevant groups

			n (%)		P
Procedure					
	Surgery after unsuccessful pneumatic reduction		39 (21.3)	A	<0.001 ^1^
	Direct surgery		29 (15.8)	B	
	Successful pneumatic reduction		115 (62.8) ^AB^	C	
İntussusception localization					
	Ileoileal		14 (7.7)	A	<0.001 ^1^
	Ileocolic		164 (89.6) ^AC^	B	
	Colocolic		5 (2.7)	C	
Gender					
	Female		67 (36.6)	A	<0.001 ^2^
	Male		116 (63.4) ^A^	B	
İntussusception with pathologic lead point					
	HSP (Henoch-Schönlein Purpura)		1 (7.1)		0.112 ^1^
	Mesenteric lymphadenitis		7 (50.0)		
	Meckel’s Diverticulum		4 (28.6)		
	Polyps		2 (14.3)		
Season of Presentation					
	Winter		42 (23.0)		0.900 ^1^
	Spring		49 (26.8)		
	Summer		47 (25.7)		
	Fall		45 (24.6)		

No difference was determined between the patients included in the study in respect of the season of presentation. While mesenteric lymphadenitis (n = 7) was determined as the most common lesion in cases where a pathologic lead point was detected (7.6%), Meckel’s diverticulum was observed in four patients, polyps in two patients, and an involvement associated with Henoch-Schönlein Purpura in one patient. It was seen that all cases with a pathologic lead point were referred directly for surgery. The pneumatic reduction procedure was applied to 154 (84.1%) patients and successful results were obtained in 115 (74.7%), while surgical intervention was required in 39 (25.3%) patients. A total of 29 (15.8%) cases were directly taken for surgery following evaluation of the clinical and imaging results. Intussusception was determined most frequently in the ileocolic (89.6%) area and the majority of cases were suitable for pneumatic reduction (p < 0.001) (Table 1).

In females, the frequency of surgery after pneumatic reduction was determined to be higher than the frequency of direct surgery (p < 0.001) (Table 2).

**Table 2:** Table comparing the patients undergoing pneumatic reduction and surgery ^1^ Pearson Chi-square test (Monte Carlo) ^2^ Fisher-Freeman-Halton test (Monte Carlo) ^3^ Kruskal-Wallis test (Monte Carlo); Post hoc test:Dunn's test ^4^ One-way ANOVA (Robust Statistic:Brown-Forsythe) ^A^ Significant according to the pneumatic reduction (-) surgery group ^B^ Significant according to the direct surgery group ^C^ Significant according to the pneumatic reduction group SD: Standard deviation; CRP: C-reactive protein; AST: Aspartate aminotransferase; ALT: Alanine aminotransferase.

		Surgery after unsuccessful pneumatic reduction		Direct surgery		Successful pneumatic reduction	P
		(n = 39)		(n = 29)		(n = 115)	
İntussusception localization							
	Ileoileal	6 (15.38)		1 (3.45)		7 (6.09)	0.258 ^2^
	Ileocolic	33 (84.62)		27 (93.10)		104 (90.43)	
	Colocolic	0 (0.00)		1 (3.45)		4 (3.48)	
Gender							
	Female	21 (53.85) ^B^		6 (20.69)		40 (34.78)	0.015 ^1^
	Male	18 (46.15)		23 (79.31) ^A^		75 (65.22)	
Age (years)							
	≤2	15 (38.5)		8 (27.6)		20 (17.4) ^A^	<0.001^2^
	3	7 (17.9)		19 (65.5) ^AC^		69 (60.0)	
	4	2 (5.1)		2 (6.9)		23 (20.0) ^A^	
	5	2 (5.1)		0 (0.0)		1 (0.9)	
	6≥	13 (33.3) ^BC^		0 (0.0)		2 (1.7)	
		Median (Min / Max)		Median (Min / Max)		Median (Min / Max)	
Duration of Surgery (min)		50 (5 / 210)		60 (10 / 120) ^C^		30 (5 / 180)	0.003 ^3^
Length of Hospital Stay (days)		5 (1 / 14) ^BC^		2 (1 / 6)		3 (1 / 9)	0.001 ^3^
CRP (mg/dL)		1.57 (0.01 / 11.81)		0.475 (0.01 / 11.75)		0.93 (0.01 / 20.88)	0.323 ^3^
AST (IU/L)		30 (17 / 262)		30 (22 / 51)		34 (16 / 113)	0.717 ^3^
ALT (IU/L)		16 (6 / 258)		14.5 (6 / 28)		16 (6 / 72)	0.294 ^3^
Urea (mg/dL)		14 (6 / 39)		19 (9.49 / 44)		18.145 (4.66 / 50)	0.094 ^3^
Creatinine (mg/dL)		0.37 (0.01 / 0.65)		0.405 (0.01 / 0.51)		0.4 (0.02 / 0.8)	0.231 ^3^
Hct (%)		33.94 (26.34 / 41.41)		35.29 (31.63 / 42.22)		35.275 (17.12 / 43.56)	0.443 ^3^
Hb (g/dL)		11.04 (8.69 / 12.83)		10.93 (9.382 / 13.22)		11.375 (6.094 / 14.23)	0.816 ^3^
		Mean ± SD		Mean ± SD		Mean ± SD	
WBC (10^6^*mm^3^)		15.13 ± 4.70		15.04 ± 5.87		13.50 ± 4.39	0.366 ^4^

In females, the frequency of surgery after pneumatic reduction in females was determined to be higher than the frequency of direct surgery (p < 0.001) (Table 2). While frequency of admission to direct surgery following the failure of pneumatic reduction rates in children under the age of two years was higher than the need to surgical intervention after unsuccessful pneumatic reduction rates (Table 2). The pneumatic reduction success rate was determined to decrease significantly in children aged ≥6 years (66.7%) (p < 0.001) (Table 2).

The mean duration of surgery of the children who underwent direct surgery because of intussusception was determined to be longer than those of other interventions (60 min) and the difference between this group and those with successful pneumatic reduction (30 min) was statistically significant (p = 0.008) (Table 2). The duration of surgery of the group who underwent surgery after unsuccessful pneumatic reduction was not observed to be longer than that of cases who underwent direct surgery.

The mean length of hospital stay of patients who underwent surgery after pneumatic reduction (five days) was significantly longer than that of patients who underwent direct surgery and for whom successful pneumatic reduction was ensured (p = 0.001 and p = 0.008, respectively). No significant difference was determined between the successful pneumatic reduction and direct surgery groups. There was no significant difference between the intervention methods and the laboratory parameters of the cases at the time of presentation (p > 0.05) (Table 2).

## Discussion

Operative and non-operative treatment options are used in the treatment of intussusception [2,7]. Non-operative reduction treatments are used as the first option in clinically suitable patients without contraindications [8-10]. Early surgery is recommended in cases where this option is not useful. Pneumatic reduction has come into routine prominent use as a method with a higher reduction rate and lower complication risks than barium enema and hydrostatic reduction interventions. The perforation risk has been reported to be 0.1-3% and the relapse risk, 6-10%, in hydrostatic and pneumatic reductions, and the relapse rate is 10% in conservative surgical treatment. The results of this retrospective study showed that although the complication of perforation was encountered, the success rate of pneumatic reduction was 74.7% (115 successful / 39 unsuccessful) and this success rate was comparable with the rates obtained in similar case series [8,11,12].

While pneumatic reduction was performed under fluoroscopy guidance in the early periods of clinical use, there has been a tendency towards USG-guided procedures in recent years to minimize radiation exposure in children. Although USG has excellent diagnostic power in the diagnosis of intussusception, it does not have the same diagnostic value as computed tomography (CT) in cases of secondary or complicated intussusception. A major limitation of this method is the requirement for experienced surgeons and radiologists. There are also studies explaining difficulties in the use of this method because of gas artifacts in the abdomen, which reduce the chance of success of USG-guided reduction [13,14]. In our clinic, US is used routinely during the diagnosis and treatment of intussusception, while fluoroscopic imaging is used for patients who cannot be differentiated during the reduction process.

In a retrospective review, Okazaki et al. reported that quite a high success rate can be achieved in the USG-guided pneumatic reduction procedure with an experienced radiologist and surgeon (approximately 94%) [15]. It was concluded that this was associated with the reduction of mortality and morbidity rates because of the chance of early intervention by the surgeon who performed the procedure if there was any newly-developed perforation or dehydration. US-guided and fluoroscopy-guided pneumatic reductions are commonly used methods in our clinic, which is staffed by experienced radiologists and surgeons. The results of the present study can be considered of value for our center as the rate of reduction success was found to be close to rates in literature and both operating time and length of hospital stay were short.

In the literature, lower success rates have been reported in non-operative reduction interventions because pathologic lead point-induced intussusception is more common in infants under two years of age [16,17]. In the present study, parallel to these data, it was determined that the frequency of surgery after unsuccessful pneumatic reduction rate in children ≤2 years of age was significantly higher than pneumatic reduction success rates (p < 0.001). This may be explained by more frequent pathological obstruction causing mechanical obstruction leading to intussusception in children under two years of age.

The performance of USG-guided hydrostatic reduction and fluoroscopy-guided pneumatic reduction procedures were compared in a randomized controlled study conducted by Xie et al. on 124 children diagnosed with intussusception [3]. In that study, the success rate of hydrostatic reduction was found to be significantly higher than that of the pneumatic reduction (96.87% vs. 83.87%), while there was no significant difference between the two procedures in terms of perforation and recurrence risks. The effect of an experienced radiology team on the success of the USG-guided reduction was emphasized in that study. In our clinic, fluoroscopy is frequently used particularly for the reduction procedure in emergency cases. Therefore, it is thought that there is a need for controlled studies to determine the success of USG-guided pneumatic reduction performed by experienced radiologists in our clinic.

Children are expected to benefit more from pneumatic reduction with increasing age. However, the present study revealed that the success rate of pneumatic reduction in children in the age range of 0-4 years was quite high and the rate decreased in children aged ≥6 years. The pneumatic reduction failure rates were higher in females compared to males and therefore, there is a need for the reasons for this to be evaluated in detail with further large-scale studies (failure rates: 34.4% in females, 19.4% in males) (p < 0.001).

In recent studies, the risk of intussusception has been shown to increase as a result of rotavirus-induced diarrhea particularly in the first four-month period of infancy and the rotavirus vaccine is recommended for infants in postnatal week 6-15 [4]. The frequency of intussusception has been shown to increase in months when the diarrhea frequency increased before the rotavirus vaccine and this condition has been seen to improve with the implementation of regular vaccination programs in recent years. In the present study, it was significant that the frequency of intussusception did not differ between seasons.

Limitations of the study

The present study had some limitations because of the retrospective nature. The strength of the study results can be considered to have been decreased due to the inability to access data related to symptom duration before presentation at the emergency service, history of abdominal pathology and recurrence rates in children determined with intussusception. In addition, the superiority of the fluoroscopy-guided pneumatic reduction procedure, which is the routine procedure in our clinic, was not evaluated. However, it was important that the results obtained in this study using the fluoroscopy-guided pneumatic reduction procedure were close to the rates reported in the literature.

## Conclusions

In conclusion, the fluoroscopy-guided pneumatic reduction procedure has a high success rate and is still one of the first option methods in the treatment of idiopathic intussusception. The operation performed under operating room conditions is more suitable for patient safety. It is thought that non-operative reduction success in patients in this group may be increased through detailed evaluation of children aged ≤2 years of age detected with a pathologic lead point.
